# An atlas of Wnt activity during embryogenesis in *Xenopus tropicalis*

**DOI:** 10.1371/journal.pone.0193606

**Published:** 2018-04-19

**Authors:** Caroline Borday, Karine Parain, Hong Thi Tran, Kris Vleminckx, Muriel Perron, Anne H. Monsoro-Burq

**Affiliations:** 1 CNRS UMR 3347, INSERM U1021, Univ. Paris Sud, Université Paris Saclay, Centre Universitaire, Orsay, France; 2 Institut Curie Research Division, PSL Research University, CNRS UMR 3347, INSERM U1021, Orsay, France; 3 Paris-Saclay Institute of Neuroscience, CNRS, Univ Paris Sud, Université Paris-Saclay, Orsay, France; 4 Department of Biomedical Molecular Biology, Ghent University, Ghent, Belgium; 5 Institut Universitaire de France, Paris, France; Laboratoire de Biologie du Développement de Villefranche-sur-Mer, FRANCE

## Abstract

Wnt proteins form a family of highly conserved secreted molecules that are critical mediators of cell-cell signaling during embryogenesis. Partial data on Wnt activity in different tissues and at different stages have been reported in frog embryos. Our objective here is to provide a coherent and detailed description of Wnt activity throughout embryo development. Using a transgenic *Xenopus tropicalis* line carrying a Wnt-responsive reporter sequence, we depict the spatial and temporal dynamics of canonical Wnt activity during embryogenesis. We provide a comprehensive series of *in situ* hybridization in whole-mount embryos and in cross-sections, from gastrula to tadpole stages, with special focus on neural tube, retina and neural crest cell development. This collection of patterns will thus constitute a valuable resource for developmental biologists to picture the dynamics of Wnt activity during development.

## Introduction

The Wnt/β-catenin pathway plays a crucial role in cell proliferation, cell polarity and cell fate determination during vertebrate development [1]. Its early deregulation in the mouse is embryonic lethal. At later development stages, abnormal Wnt/*β*-catenin signaling results in birth defects. In adults, Wnt/*β*-catenin signaling deregulation leads to cancer and other diseases [2]. Intense research seeks to better understand Wnt signaling and to develop therapies for the treatment of tumors.

Wnt proteins are secreted by the signaling cells, diffuse over short or long range [3] and act on target cells through either β-catenin-dependent or -independent Wnt pathways (reviewed in [4]). In the former case, when a given Wnt ligand binds to its cognate Frizzled receptor(s) and LRP5/6 co-receptors, this results in a complex intracellular cascade, leading to β-catenin stabilization. β-catenin then translocates into the nucleus, associates with LEF/TCF family transcription factors and induces the transcriptional activation of Wnt target genes such as *CyclinD1* and *Axin2* [5,6].

The developmental expression of Wnt/*β*-catenin pathway components has been extensively described in vertebrate animal models, including expression of various Wnt ligands and Wnt receptors (reviewed in [4]). However, multiple extracellular, cytoplasmic, and nuclear inputs are integrated and modulate Wnt signaling. For example, receptor-ligand specificity and multiple feedback loops control Wnt signaling efficiency (reviewed in [4]). Additionally, it was recently described in zebrafish that Wnt8a can be transported over long distances within the signaling cell through filopodia, increasing Wnt signaling range [7]. This makes it difficult to infer the Wnt responsive tissue from the site of ligand synthesis. Another way to describe Wnt activity is to study the expression of known direct target genes. However, *CyclinD1*, for instance, is transcriptionally regulated by many other inducers and repressors (reviewed in [8,9]) and thus is not a strict readout for Wnt activity. *Axin2*, also widely used as reporter, is not fully reliable either: for example it is found not to be expressed in mouse lung cells while Wnt/*β*-catenin pathway is active in these cells [10].

Finally, it is possible to follow Wnt/*β*-catenin activity by using transgenic lines allowing monitoring the spatial and temporal activity through the expression of a reporter gene (*gfp* or *lacZ*). The majority of these lines are generated in mice (Table 1). These lines often rely on the expression of a reporter gene driven by a Wnt target gene promoter. However, these lines may be questionable since the reporter gene expression differs significantly between different reporter mice [10] probably due to specific regulation of each promoter.

**Table 1:** Wnt reporter transgenic lines in vertebrates.

Transgenic line names	Species	References
TOP-GALC	mouse	[11]
ins-TOPEGFP and ins-TOPGAL	mouse	[12]
Lgr5-EGFP-IRES-creERT2	mouse	[13]
LEF-EGFP	mouse	[14]
TCF/Lef:H2B-GFP reporter TCF/Lef-LacZ	mouse	[15]
Axin2-CreERT2	mouse	[16]
Tcf3-CreER	mouse	[17]
p-LEF_7_-fos-GFP	Xenopus	[18]
TOP/FOPTK-iGFP	Xenopus	[19]
pbin7Lef-dGFP	Xenopus	[20]
TOPdGFP	zebrafish	[21]
Tcf/Lef-miniP:dGFP	zebrafish	[22]
*Tg(7xTCF-Xla*.*Siam*:*GFP)*^*ia4*^ and *Tg(7xTCF-Xla*.*Siam*:*nlsmCherry)*^*ia5*^	zebrafish	[23]

To obtain a more direct and reliable readout for Wnt/β-catenin signaling, we here use a synthetic promoter harboring seven optimal binding sequences for LEF-1/TCF [24]. A transgenic reporter line, in which *gfp* gene expression is driven by this synthetic promoter, was generated in the frog *Xenopus tropicalis*, allowing visualization of Wnt/β-catenin activity *in vivo*. The line was validated previously as a reliable tool to monitor Wnt activity in tadpoles treated with compounds known to modulate Wnt activity: activation with 6-bromoindirubin-3-oxime (BIO), a selective GSK-3 inhibitor [25], or inhibition with IWR-1, a small molecule that prevents Axin protein degradation [26], [27]. This transgenic line, was previously used to study Wnt activity during eye or brain development [27–29], allows generating many transgenic embryos with reproducible *in vivo* expression patterns [20].

Here, we provide a detailed atlas illustrating Wnt/β-catenin spatio-temporal activity during *Xenopus tropicalis* embryogenesis, using whole-mount *in situ* hybridization and serial transverse sections at various developmental stages, from gastrula (stage 11) to tadpole (stage 40) stages. We provide a complete collection of pictures in supplementary data (Figs A-Q in S1 File). Moreover, we provide in-depth analysis of Wnt activity during three selected developmental processes: neural tube patterning, neural crest specification and migration and retinal development. We take advantage of this study to compare our observations with the data scattered in various previous articles.

## Materials and methods

### Ethics statement

Animal care and experimentation were conducted in accordance with institutional and national guidelines, under the institutional licenses (number B 91-471-102 up to 2012 and C 91-471-102 since 2013). Protocols were approved by the “Comité d’éthique en experimentation animale n°118” and received an authorization by the “Ministère de l’Education Nationale, de l’Enseignement Supérieur et de la Recherche” under the reference APFIS#7043.

### Embryos

*Xenopus tropicalis* transgenic embryos were obtained by conventional methods of hormone-induced egg laying and *in vitro* fertilization [30] between a wild type female and a transgenic male *Tg(pbin7Lef-dGFP)*, carrying the Wnt reporter previously described (Image A in S1 Fig; [20,24]). Beforehand, the male was selected as having a single insertion site of the transgene (as inferred by mendelian ratios in its progeny) in order to insure homogeneous levels of *gfp* expression in the offspring [27]. Embryos were grown, collected and fixed in 4% paraformaldehyde (PFA) from embryonic stage 11 to stage 40 according to Nieuwkoop and Faber’s staging table of development [31]. The embryos were then washed in 1x PBS, dehydrated in 100% methanol, and stored at –20 °C.

### *In situ* hybridization and sectioning

Digoxigenin-labeled antisense RNA probes were generated according to the manufacturer’s instructions (DIG RNA Labeling Mix, Roche) from the following plasmids: *enr2* [32], *fezf2* [33], *krox20* [34], *pax3* [35], *otx2* [36], *snai2* [37], *sox2* [38], *twist* [39] and *wnt1* [37]. A digoxigenin-labeled antisense RNA probe and a fluorescein-labeled antisense RNA probe (fluorescein-12-UTP, Roche) were generated from the plasmid *pCS2-MT-eGFP* (a gift from David Turner, University of Michigan, Ann Arbor, USA). For embryos under NF stage 20, single whole-mount *in situ* hybridization (WISH) was carried out as previously described [40]. For later stages, WISH was carried out following a protocol described by Parain et al. [41], except for the bleaching treatment that we performed after embryo staining. Briefly, following overnight incubation with the probe and then with alkaline phosphatase-conjugated anti-DIG antibody, enzymatic activity was revealed using NBT/BCIP substrate. Of note, the described patterns were observed in all the examined embryos (n≥8 for each probe). For double *in situ* hybridization, DIG-labeled probes were revealed with NBT/BCIP substrates and the fluorescein-labeled *gfp* probe was revealed with Fast Red substrate (Roche). After the first revelation, the embryos were treated by 10mM EDTA in PBS for 30 minutes at 60°C, then in 0.1M Glycine-HCl, at pH 2.2 for 10 minutes at RT, then processed for the second revelation. Sections (40μm thick) were cut using a Leica VT1000 vibratome after gelatin-albumin embedding. Sections were mounted in glycerol. The same embryo has been used to generate all of the pictures provided at a given stage (whole mount and sections).

### Microscopy

Whole-mount images were captured using a stereomicroscope Lumar V12 equipped with bright field and color camera (Zeiss). Pictures of sections were captured using a digital Axiocam MRc camera on a Leica microscope and processed with AxioVision REL 7.8 and Adobe Photoshop CS4 softwares.

## Results and discussion

### An atlas of Wnt activity during development

**Fig 1:**
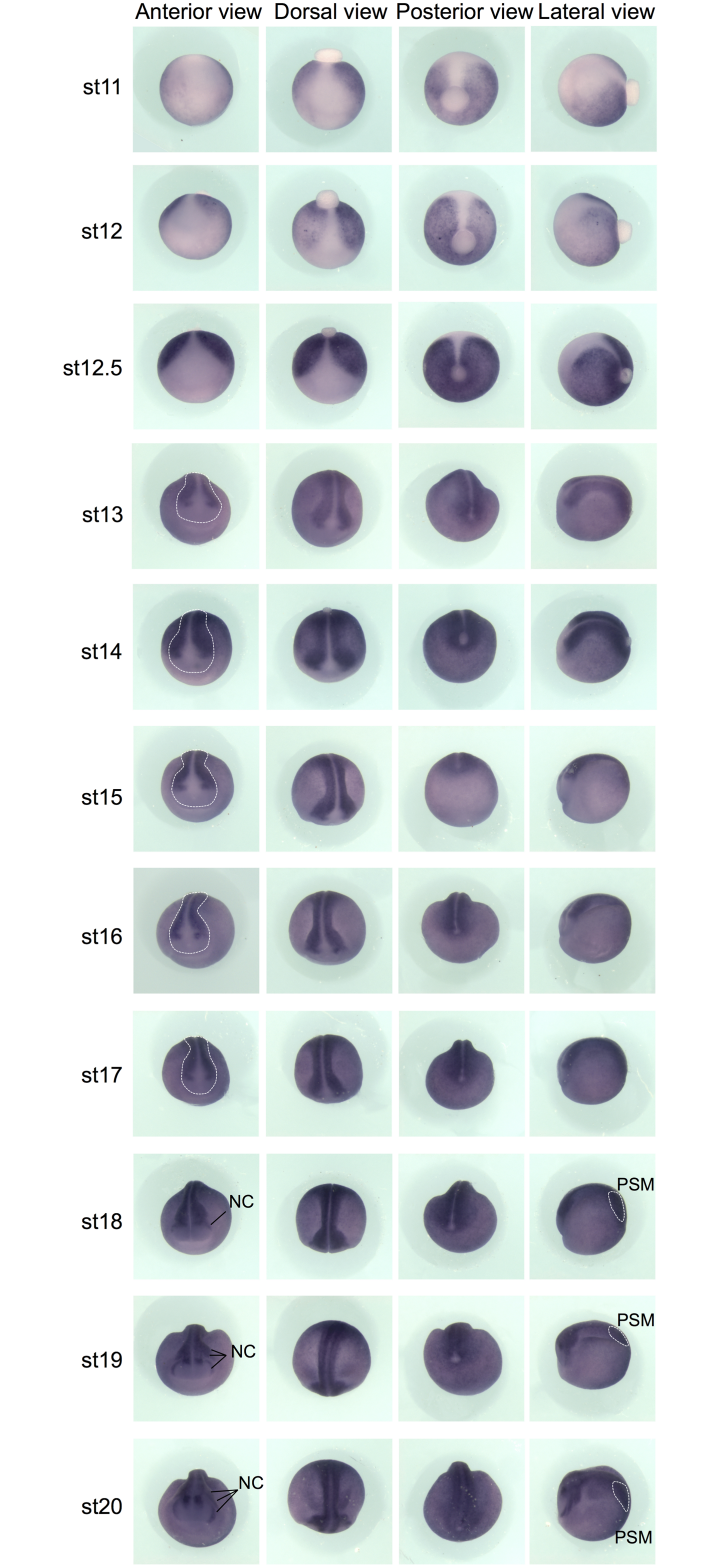
Wnt activity during gastrulation and neurulation in whole embryos. Whole-mount *gfp in situ* hybridization on Tg(pbin7Lef-dGFP) embryos from stage 11 to stage 20. For each stage, anterior, dorsal, posterior and lateral views are shown. White dotted lines on anterior views delineate the prospective central nervous system during neurulation. NC: migrating neural crest cells, PSM: posterior presomitic mesoderm.

**Fig 2:**
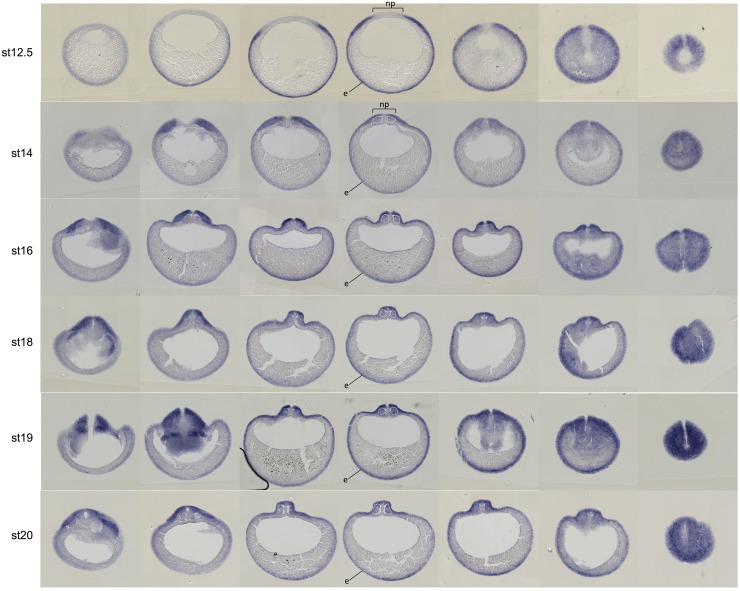
Wnt activity during gastrulation and neurulation in cross-sections. Embryo transverse (or cross-)sections following whole-mount *gfp in situ* hybridization on Tg(pbin7Lef-dGFP) embryos, from stage 12.5 to stage 20. For each stage, 7 serial sections are shown. White dotted lines delineate the somites. e: nonneural ectoderm; np: neural plate: brackets indicate the approximate width of the neural plate.

**Fig 3:**
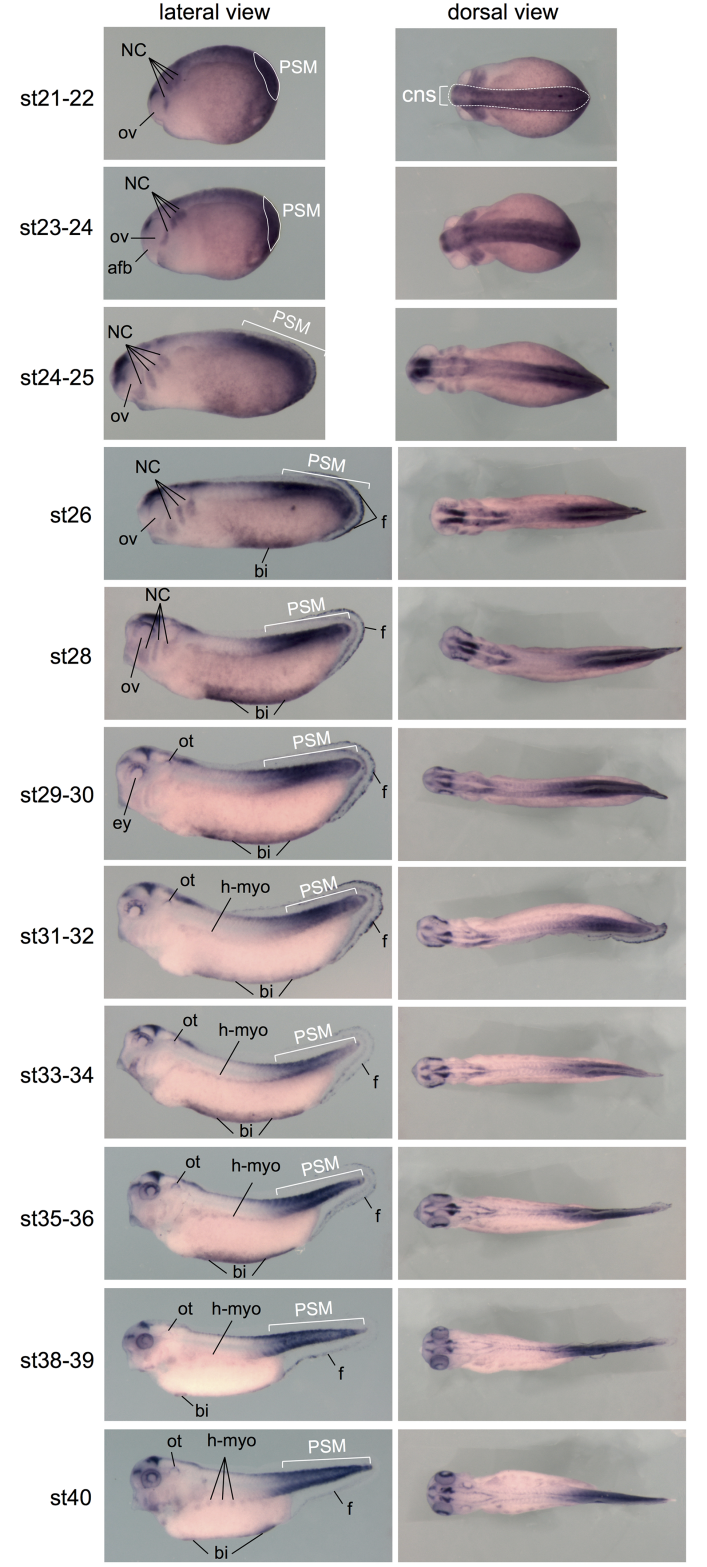
Wnt activity during organogenesis in whole embryos. Whole-mount *gfp in situ* hybridization on Tg(pbin7Lef-dGFP) embryos from stage 21 to stage 40. For each stage, lateral (side) and dorsal views are shown afb: anterior part of the forebrain; bi: blood islands; cns: central nervous system; ey: eye; f: fins; h-myo: hypaxial myoblast; NC: migrating neural crest cells; ot: otic vesicle; ov: optic vesicle; PSM: posterior presomitic mesoderm.

**Fig 4:**
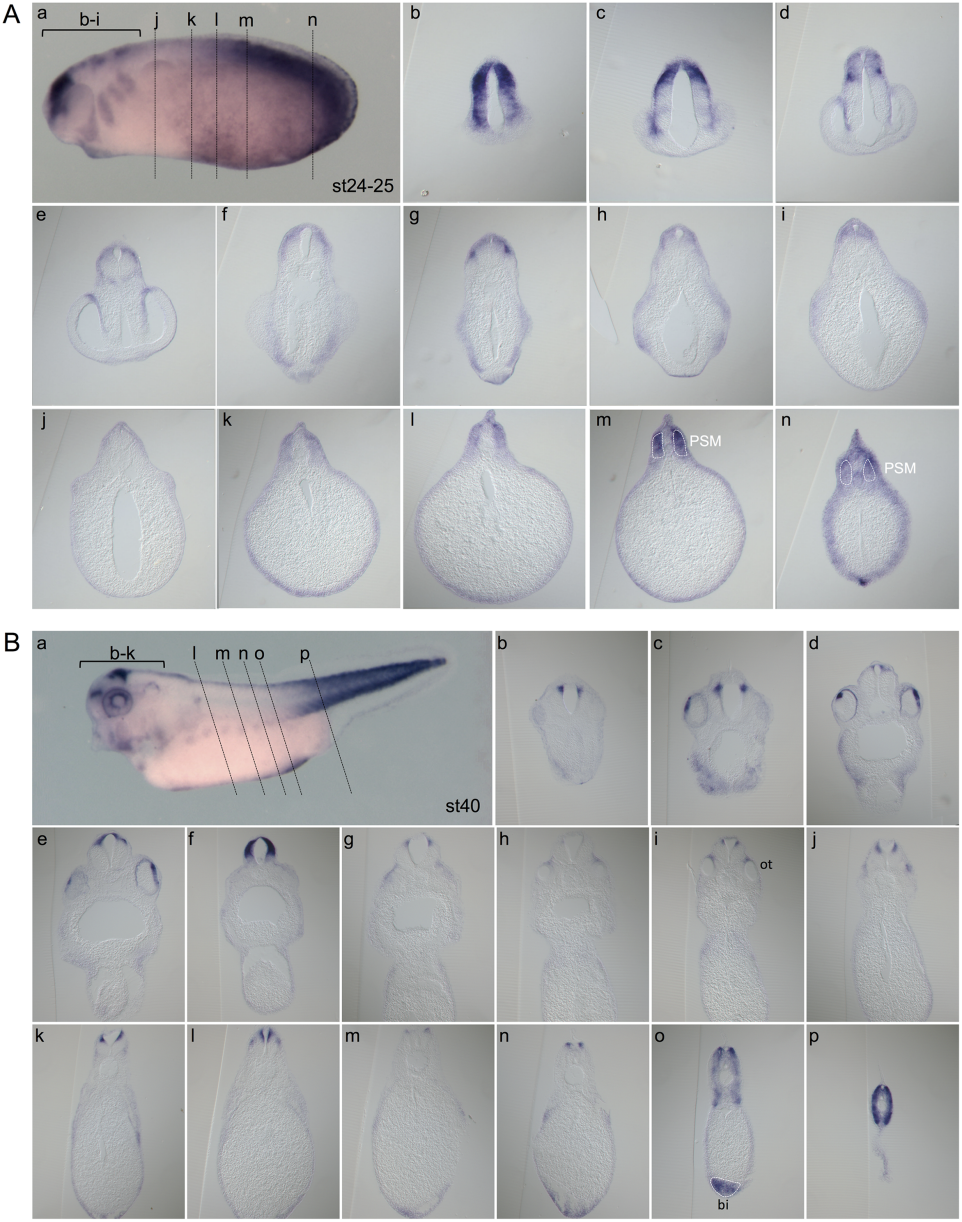
Wnt activity at stages 24–25 and 40. Whole-mount *gfp in situ* hybridization (Aa, and Ba, lateral views) of Tg(pbin7Lef-dGFP) embryos at stage 24–25 **(A)** and stage 40 **(B)**. For each stage, transverse sections are shown (Ab-n and Bb-p). The different levels of sections are indicated in panels a. bi: blood islands; ot: otic vesicle. PSM: posterior presomitic mesoderm.

To provide an atlas depicting canonical Wnt/β-catenin activity during embryogenesis, we took advantage of the transgenic *Xenopus tropicalis* Wnt reporter line, *Tg(pbin7Lef-dGFP)*, described in [20] (Image A in S1 Fig). Briefly, the transgene, flanked by chromosomal insulator sequences derived from the chicken ß-globin locus, contains a synthetic promoter harboring seven copies of an optimal binding sequence for LEF-1/TCF upstream of eGFP coding sequence. Because a weak GFP fluorescence signal can be difficult to distinguish from the natural auto-fluorescence of the embryos, and to obtain a clear staining in tissue with low levels of expression, we used whole-mount *in situ* hybridization with a *gfp* antisense probe to detect the Wnt/β-catenin activity. From gastrula (stage 11) to tadpole (stage 40) stages, whole-mount-stained embryos were pictured from different views (anterior, dorsal, posterior and lateral) and serial transverse vibratome sections were then cut (Figs 1–4, Figs A-Q in S1 File).

During gastrulation (stage 11 to 12.5), *gfp* transcripts are detected around the whole embryo except in the anterior and dorsal region (Fig 1). Transverse sections at stage 12.5 show a staining restricted to the inner ectodermal cell layer called “sensory" (or basal) layer of the non-neural ectoderm (Image B in S1 Fig). This is consistent with *Xenopus* experiments illustrating that Wnt/β-catenin signaling regulates specification and differentiation of cells in *Xenopus* mucociliary epidermis ([42], reviewed in [43]).

From the end of gastrulation (stage 13) onwards, Wnt activity is detected in the developing central nervous system, except in its anterior-most region (Fig 1, dotted lines). From late neurula stage (stage 18) onwards, *gfp* transcripts become particularly abundant in the migrating neural crest cells. The canonical Wnt/β-catenin pathway is also strongly active in the posterior presomitic mesoderm (Fig 1). This is consistent with data showing nuclear β-catenin translocation during maturation of this structure in mouse [44]. On sections, we observe a faint staining in the somites at stage 14 (Fig 2, white dotted lines). The somite staining increases to reach a strong level at stage 20. Previous studies in chick and mouse have shown that Wnt signaling promotes the dermomyotome fate and not the sclerotome fate during somite patterning [45,46]. In *Xenopus*, the majority of somite cells express *myoD* indicating that they are almost all myotome cells [47]. Consistently, we show here that somites are entirely Wnt-responsive (Fig 2 and Image C in S1 Fig). From stage 21, *gfp* mRNAs are detected in the central nervous system, in the neural crest cells migrating towards the branchial arches, and in the periphery of the optic vesicle (Fig 3). These patterns will be described in the following paragraphs. Until the tadpole stage, we observe that the canonical Wnt/β-catenin pathway is still strongly active in the posterior presomitic mesoderm (Figs 3, 4Am and 4An). From stage 31–32, a weak Wnt activity is detected in myotome cells that have emigrated from the somite to form hypaxial muscles (Fig 3). From stage 29–30, expression in the otic vesicles is also apparent, and persists until tadpole stage (Fig 3). The staining is restricted to the dorsal part of the otic vesicle, as confirmed on sections (Fig 4Bi). We can also note that, whereas ventral and dorsal *Xenopus* fins have independent induction and formation processes [48], they are both Wnt-responsive from stage 26 to stage 31–32 (Fig 3 and Image D in S1 Fig). Finally, Wnt/β-catenin activity is also detected in the ventral blood islands from stage 26 (Figs 3 and 4Bo), where its role on specification and maintenance of the primitive blood cells has been demonstrated [24].

### Wnt activation during neural tube development

**Fig 5:**
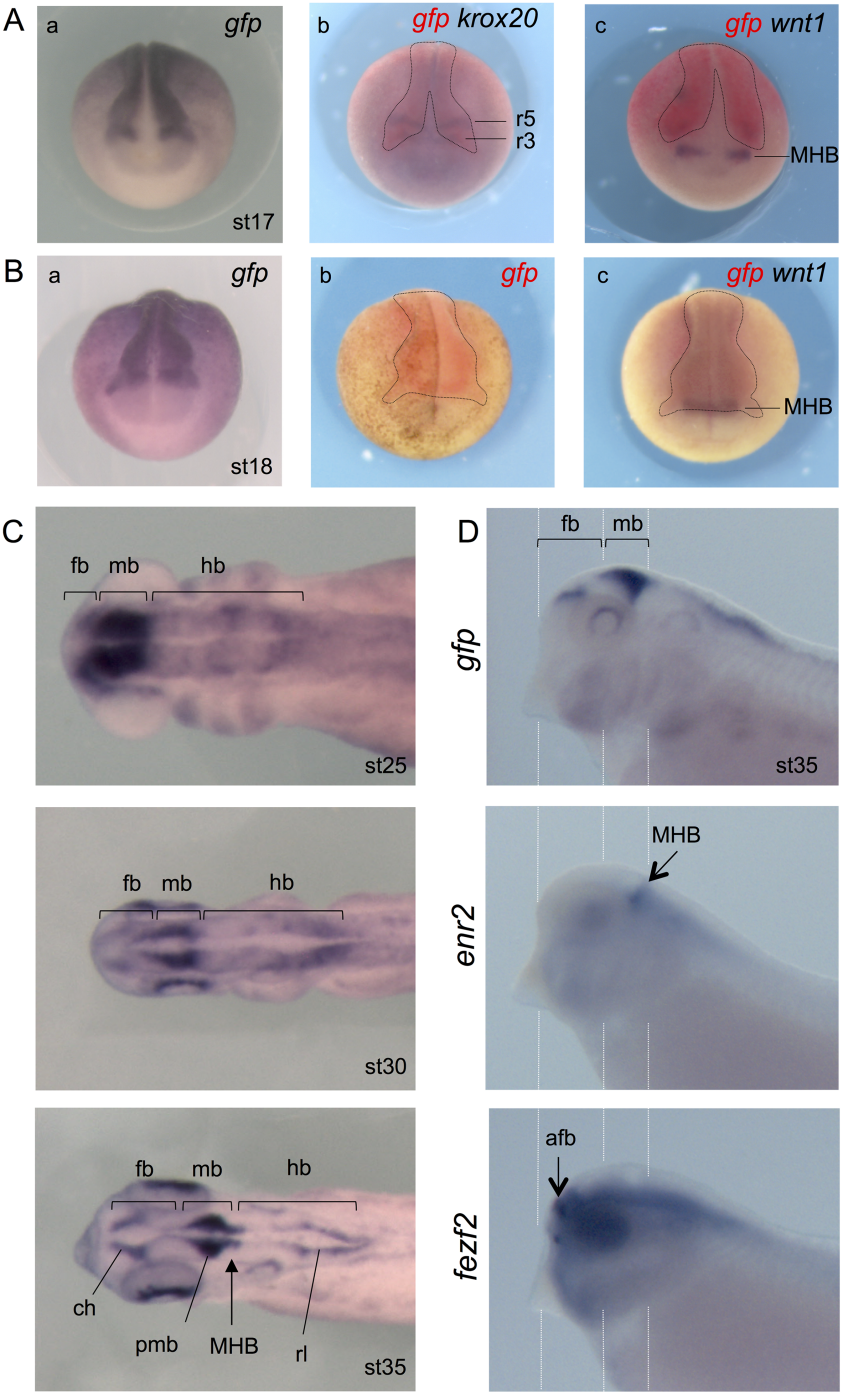
Wnt activity during neural tube development. **(A)** Anterior views of *Tg(pbin7Lef-dGFP)* embryos at stage 17 hybridized with probes against *gfp* alone (a) or *gfp* and *krox20* or *gfp* (b) or *gfp* and *wnt1(c)*. Dotted lines delineated the *gfp* staining. **(B)** Anterior views of *Tg(pbin7Lef-dGFP)* embryos at stage 18 hybridized with probes against *gfp* alone revealed with NBT/BCIP (a) or Fast Red (b) substrates or *gfp* and *wnt1* (c). The same embryo is shown in b and c. Dotted lines delineate the *gfp* staining. **(C)** Dorsal views of embryos hybridized with probe against *gfp* at stage 25, 30 and 35. **(D)** Lateral views of the anterior part of embryos hybridized with probes against *gfp*, *fezf2* or *enr2* at stage 35. afb: anterior forebrain; ch: cortical hem; fb: forebrain; hb: hindbrain; mb: midbrain; MHB: Midbrain Hindbrain Boundary; pmb: posterior midbrain; r3 and r5: rhombomeres 3 and 5; rl: rhombic lip.

*In situ* hybridization at stage 12.5 and 13 shows an exclusion of *gfp* transcripts from most of the anterior neural plate (Fig 2), as confirmed by double *in situ* hybridization with the neural plate marker *sox2* (Fig 1 and Image E in S1 Fig). This is consistent with the well-known role of Wnt signaling on posteriorization of the neuroectoderm [49]. On sections at stage 14, we observe the absence of Wnt activity in the medial region of the neural plate (Fig 2). From the end of neurulation (stage 18), Wnt activity is detected in the dorsal and dorsal-lateral part of the neural tube and is excluded from its ventral-lateral part and from the floorplate (Image F in S1 Fig). During neurulation, we observe a shift of Wnt activity towards the anterior region. At stage 17, the anterior boundary of the staining co-localizes with *krox20* at the level of the rhombomere 3 and is posterior to the expression pattern of *wnt1*, a marker of the midbrain/hindbrain boundary (MHB) (Fig 5A). At stage 18, the anterior limit of Wnt activity corresponds to the *wnt1* domain (Fig 5B).

At stage 21–24, Wnt signaling is active all along the developing central nervous system, except in the more anterior region, i.e. located anterior to the optic vesicles and corresponding to the anterior part of the forebrain (Fig 3). From stage 25, discontinuities in the staining appear in the central nervous system, some areas being less labeled than others (Fig 5C). We observe a very high *gfp* expression in the midbrain and at least a part of the forebrain. In the hindbrain, domains with high or low *gfp* expression alternate, consistent with the role of the Wnt signaling in the zebrafish hindbrain metamerization [50]. Later, from stage 30, the staining in the brain is more restricted. We observe Wnt activity in the posterior region of the midbrain, which seems to be just anterior to the MHB as suggested by the comparison with the engrailed (*enr2*) mRNA hybridization, a MHB marker (Fig 5D). Moreover, *gfp* transcripts become detectable in a brain region derived from the forebrain. This *gfp* positive region seems to partially co-localize with *fezf2* expression pattern, a marker of the anterior part of the diencephalon (prosomere p3) and of the telencephalon [51] (Fig 5D). This region is probably the cortical hem, an organizing center in the telencephalon known to present enriched expression of multiple members of the Wnt morphogens [52–54]. The cortical hem gives rise to the sub-ependymal zone where reside adult neural stem cells in rodents and human. At the level of the hindbrain, Wnt activity is detected dorsally, in the lower rhombic lip (Fig 5C). Rhombic lip produces the granular neuron progenitors of the cerebellum. By using Wnt reporter mice, it has been shown that Wnt/*β-catenin* activity is present transiently at the embryonic rhombic lip during development of the mouse cerebellum [55]. Both the rhombic lip and cortical hem are germinal zones where neurogenesis takes place and neurons are distributed tangentially.

### Wnt activity during neural crest specification/migration

Neural crest is a migratory cell population, which gives rise to many cell types such as neurons and glia of the peripheral nervous system, pigment cells, and progenitors of craniofacial mesenchyme and skeleton. These cells are specified at the border between the neural and nonneural ectoderm, an area named the neural (plate) border.

**Fig 6:**
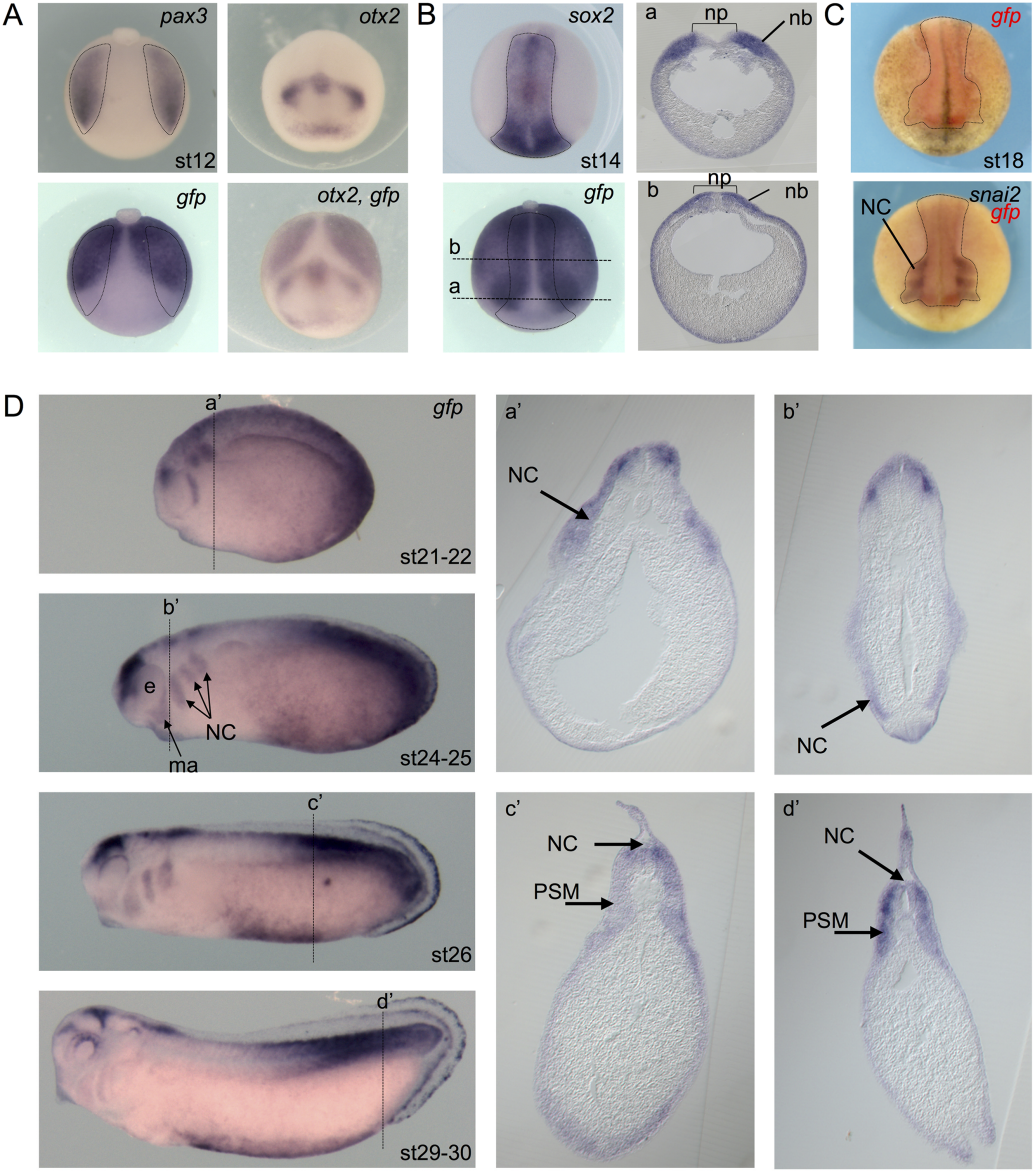
Wnt activity during neural crest formation. **(A)** Dorsal view of a stage 12 *Tg(pbin7Lef-dGFP)* embryo hybridized with *pax3 or gfp* probes (posterior side is up). Dotted shapes delineate the presumptive neural border on both sides. Anterior view of a stage 12 *Tg(pbin7Lef-dGFP)* embryo hybridized with *otx2* probe alone or *otx2* and *gfp* probes together (dorsal side is up). (**B)** Dorsal view of a stage 14 *Tg(pbin7Lef-dGFP)* embryo hybridized with *sox2* or *gfp* or *both* probes. Dotted shapes delineate the neural plate. The a and b dotted lines indicate the level of shown transverses sections. **(C)** Anterior views of a stage 18 *Tg(pbin7Lef-dGFP)* embryo first hybridized with probe against *gfp* and secondarily with probe against *snai2*. Dotted lines delineate *gfp* staining. **(D)** *In situ* hybridization against *gfp* on stage 21–22, 24–25, 26 and 29–30 *Tg(pbin7Lef-dGFP)* embryos (lateral views). For each stage, the dotted line indicates the level of the shown transverse section (a’-d’). ma: mandibular arch; nb: neural border; NC: migrating neural crest cells; np: neural plate; PSM: posterior presomitic mesoderm.

At stage 12, by using *pax3* expression pattern (dotted lines on Fig 6A) to define the neural border, we observe that Wnt activity is present in the neural border except in its most anterior part [56]. This observation is confirmed by double *in situ* hybridization (Image G in S1 Fig). These data are consistent with the described involvement of Wnt signaling in posterior neural border specification [57–59]. In parallel, Wnt activity is excluded from the *otx2*-expressing domain, which labels the anterior part of the brain (Fig 6A and Image G in S1 Fig).

In the early neurula (stage 14), Wnt activity is detected in the entire neural border and lateral part of the neural plate (expressing *sox2)*, excluding only the ventral neural plate, as confirmed on sections (Fig 6B). This is consistent with the Wnt role in neural crest specification [60–63].

From late neurula stage (stage 18), *gfp* transcripts are abundant in the migrating neural crest cells, as observed by comparing *gfp* expression pattern with that of *snai2*, a neural crest marker (Fig 6C). This location of the Wnt/β-catenin activity was recently described also in the chick [64]. Interestingly, the anterior boundary of the *gfp* staining corresponds to the more anterior migrating neural crests cells. At stage 24–25, Wnt activity is clearly detected in neural crest cells surrounding the eye, in the mandibular arch, and in the 3 following branchial arches (Fig 6D). This staining in the branchial arches disappears at stage 31–32, as neural crest cells reach their destination. A number of reports from different laboratories, using *frog*, zebrafish and chick embryos, have demonstrated that distinct elements of a non-canonical Wnt signaling, the PCP signaling, are essential for neural crest migration (reviewed in [65]). The activation of PCP signaling occurs at the cell–cell contact where it leads to the localized regulation of Rho and Rac proteins mediating directional migration of neural crest cells by controlling the formation of protrusions. Our observations suggest that Wnt signaling plays a role during neural crest cell migration not only through its PCP pathway but also through its β-catenin pathway. This is consistent with a recent paper demonstrating, through a combination of *in vitro* and *in vivo* approaches, that canonical Wnt activity is involved in neural crest migration and needs to be tightly controlled to enable it [66]. In the mouse head and branchial arch region, canonical Wnt activity is detected thanks to reporter transgenic lines in cranial neural crest cells at the neural folds, as well as in cells migrating into the face and branchial arches [14,15]. In zebrafish, interfering with LRP5 function, a co-receptor in canonical Wnt signaling, leads to a migration defects of neural crest cells [67]. Interestingly, it has been recently demonstrated in chick that the neural crest delamination requires cell-autonomously transient inhibition of Wnt signaling which needs to be reversible [64].

### Wnt signaling and retinogenesis

Wnt signaling pathway is known to regulate many aspects of retinogenesis, including patterning, specification, proliferation, regeneration, but some of these functions appear to be species specific (reviewed in [68] and [69]). We previously examined Wnt activity in the *Xenopus* retina using both *X*. *laevis* and *X*. *tropicalis* transgenic reporter lines, in different contexts such as the determination of optimal concentrations and exposure conditions of pharmacological compounds or gene expression comparison [27,29,70]. Scattered data reporting retinal Wnt activity in *Xenopus* can thus be found in these studies at different stages of eye development. In order to provide a global view of Wnt activity during retinal development in a single set of data easily explorable by the community, we decided to describe in this atlas Wnt activity at all key stages of retinogenesis in both whole-mount embryos and in retinal sections.

**Fig 7:**
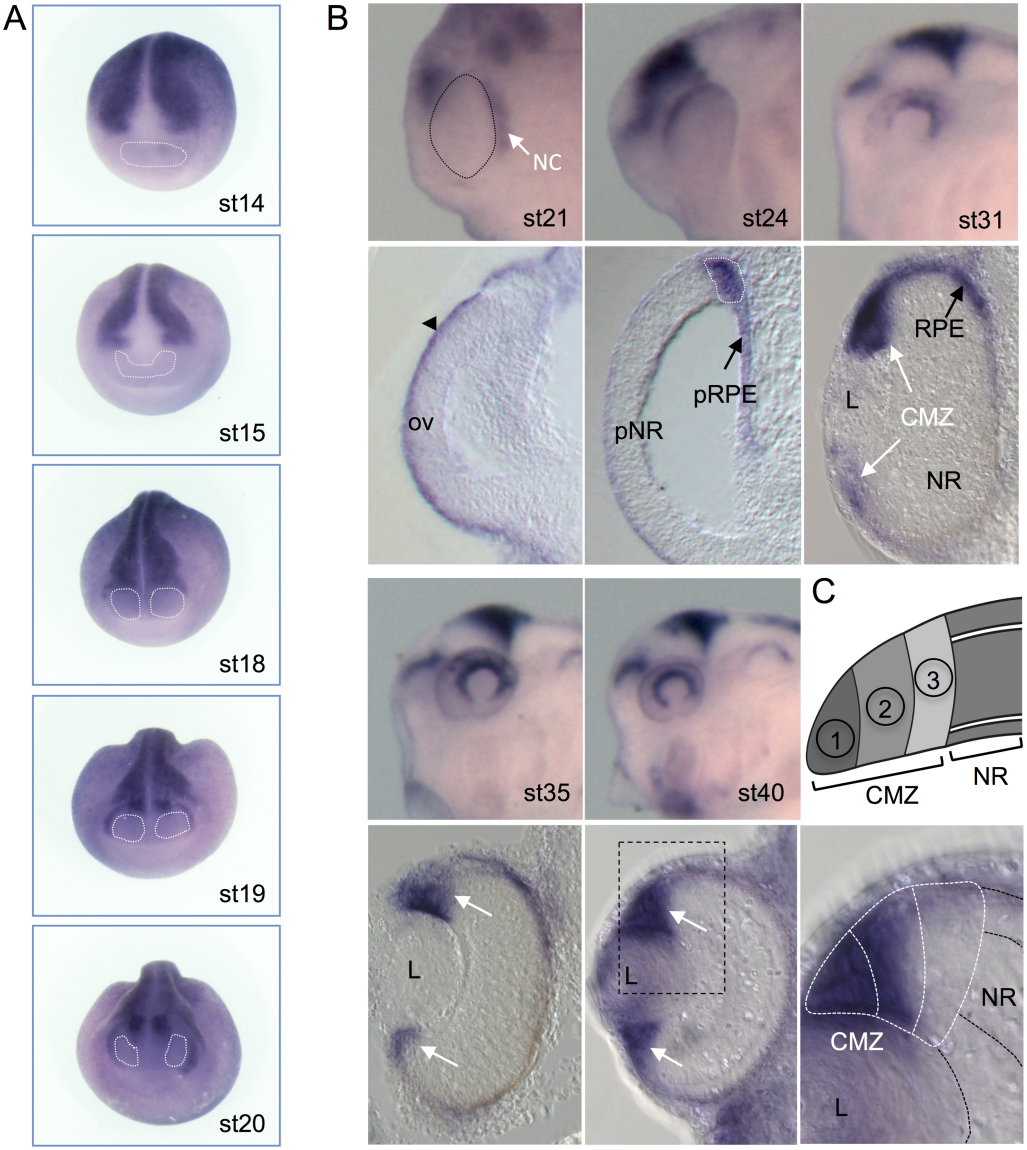
Wnt activity during retinogenesis. **(A)** Whole-mount *gfp in situ* hybridization on *Tg(pbin7Lef-dGFP)* embryos from stage 14 to stage 20. Anterior views with the eye fields (dotted lines) are shown. (B) *In situ* hybridization against *gfp* on stage 21 to stage 40 *Tg(pbin7Lef-dGFP)* embryos. Whole-mount (lateral views of the head, anterior to the left) and transverse retinal sections are shown. Arrowhead points to surface ectoderm, black arrows to the Retinal Pigmented Epithelium (RPE) and presumptive RPE (pRPE), white arrows to the Ciliary Marginal Zone (CMZ). Black dotted lines delineate the optic vesicle. White dotted lines delineate the boundary between neural *versus* non-neural territories in the retina. (C) Schematic of a CMZ showing its spatial organisation with stem cells closest to the periphery (region 1), proliferative retinoblasts in the middle (region 2) and postmitotic cells at the central edge (region 3). On the bottom, an enlargement of the region delineated with black dotted lines in the stage 40 retinal section image shows the *gfp* signal in the peripheral half of the CMZ. White dotted lines delineate the 3 zones depicted in the schema. A strong staining is observed in zone 1, a fainter staining is detected in zone 2 and barely no staining is observed in zone 3. CMZ: Ciliary Marginal Zone; L: lens; NC: migrating neural crest cells; ov: optic vesicle; pNR: presumptive Neural Retina, pRPE: presumptive Retinal Pigmented Epithelium.

We do not detect any Wnt activity during gastrulation in the eye field (doted lines in the Fig 7A). This is consistent with data demonstrating that the eye field specification requires Wnt/ β-catenin signaling inhibition [71]. Nevertheless, several evidences indicate involvement of the non-canonical Wnt signaling, resulting in the expression of different Wnt pathway components in the eye field (Wnt11, fzd5) [72]. During neurulation, the eye field splits and evaginates laterally to form the optic vesicles. Again, Wnt reporter activity is not detectable in the forming optic vesicles (Fig 7B, stage 21). However, a signal can be detected in these optic vesicles from stage 24 onwards. It is located in the presumptive retinal pigmented epithelium (pRPE) and in the most dorsal part of the optic vesicle. Active Wnt/β-catenin signaling is detected in the pRPE not only in frogs but also in chickens, fishes, and mice [21,73–75]. The spatial and temporal regulation of Wnt/ β-catenin signaling has been shown to be essential for development of the RPE in mice [76–78] and chick [79]. The restricted activation of canonical Wnt signaling in the dorsal part of the optic vesicle has also been reported in other species [73–75,79]. Our observation is consistent with the hypothesis that Wnt/β-catenin activity plays a crucial role in the maintenance of dorsal retinal identity [75,78]. It was further suggested in mouse that this dorsal Wnt activity is involved in the establishment of the proper boundary between neural *versus* non-neural territories in the retina [80]. This region in *Xenopus* has been proposed to give rise to adult retinal stem cells [29].

At stages 31 and 35, Wnt activity labeling remains visible in the developing RPE. Signal is also detected in the most peripheral part of the neural retina, the forming ciliary marginal zone (CMZ) (Fig 7B and 7C). This region contains retinal stem and progenitor cells allowing for retinal growth throughout the animal life [81]. In the mature retina (stage 40), Wnt activity is confined to the peripheral half of the CMZ where stem cells and young progenitors reside. Of note, the staining is often stronger in the dorsal side of the CMZ, consistent with the dorso-ventral gradient observed in whole-mount embryos, with no or very low staining in the most ventral side at the position of the optic fissure. We previously showed that Wnt activity in the post-embryonic CMZ is essential for retinal stem cell proliferative maintenance [27,70]. Other LEF/TCF reporters revealed activation of the Wnt/β-catenin pathway in the ciliary margin of other species [73,74]. Moreover, in mouse and chick, Wnt signaling has been described to be involved in development of this region [82,83].

To summarize, here we present a detailed atlas illustrating Wnt/*β-catenin* activity in *Xenopus tropicalis* from gastrula to tadpole stages. This library of serial pictures allows to analyse spatial and temporal activity of Wnt pathway and thereby to predict new roles of this signaling pathway. We observe that Wnt/β-catenin pathway is active in many structures during development and especially in proliferative zones of the central nervous system such as the rhombic lip, the cortical hem and the retinal ciliary marginal zone. It is also active in non-neural tissues and interestingly in the sensory layer of the epidermis, which will give rise to the stratum germinativum where stem cells will reside.

## Supporting information

S1 File Serial transversal sections from *Tg(pbin7Lef-dGFP)* embryos at different stages following whole-mount *gfp in situ* hybridization. **(Fig A)** stage 12.5, **(Fig B)** stage 14, **(Fig C)** stage 16, **(Fig D)** stage 18, **(Fig E)** stage 19, **(Fig F)** stage 20, **(Fig G)** stage 21–22, **(Fig H)** stage 23–24, **(Fig I)** stage 24–25, **(Fig J)** stage 26, **(Fig K)** stage 28, **(Fig L)** stage 29–30, **(Fig M)** stage 31–32, **(Fig N)** stage 33–34, **(Fig O)** stage 35–36, **(Fig P)** stage 38–39, **(Fig Q)** stage 40. The same embryo has been used to generate all of the images provided at a given stage.
(ZIP)

S1 Fig Focus on selected structures. **(A)** Schematic of the Wnt reporter construct containing chicken β-globin insulators [20]. **(B, C)** Embryo transverse sections following whole-mount *gfp in situ* hybridization on *Tg(pbin7Lef-dGFP)* embryos, at stage 12.5 (B) and 22 (C). Right panels are enlargement of blue squares. Black dotted lines delineate the somites. **(D)** Whole-mount *gfp in situ* hybridization of Tg(pbin7Lef-dGFP) embryos at stages 29–30 and transversal sections at indicated level showing *gfp* staining in the dorsal and ventral fins. **(E)** Dorsal views of a stage 13 *Tg(pbin7Lef-dGFP)* embryo first hybridized with probe against *gfp* and secondarily with probe against *sox2*. Black dotted lines delineate the *gfp*-expressing domain. **(F)** Embryo transverse sections following whole-mount *gfp in situ* hybridization on *Tg(pbin7Lef-dGFP)* embryos, at stage 18 and 24–25. The squared regions delineated with the dotted line were enlarged. White dotted lines delineate the neural tube. **(G)** Dorsal view of a stage 12 *Tg(pbin7Lef-dGFP)* embryo hybridized with *gfp* and *pax3* probes or *gfp* and *otx2* probes (posterior side is up). Dotted lines delineate *gfp* staining. d-f: dorsal fins; ectod.: ectoderm; ins: fp: floorplate; insulator; mesod.: mesoderm; n: notochord; np: neural plate; NT: neural tube; v-f: ventral fins.
(TIF)
