# Supplementary figures and images for: An atlas of Wnt activity during embryogenesis in Xenopus tropicalis
Source: PLoS One. 2018 Apr 19;13(4):e0193606. doi: 10.1371/journal.pone.0193606 (PMC5908154; doi:10.1371/journal.pone.0193606)

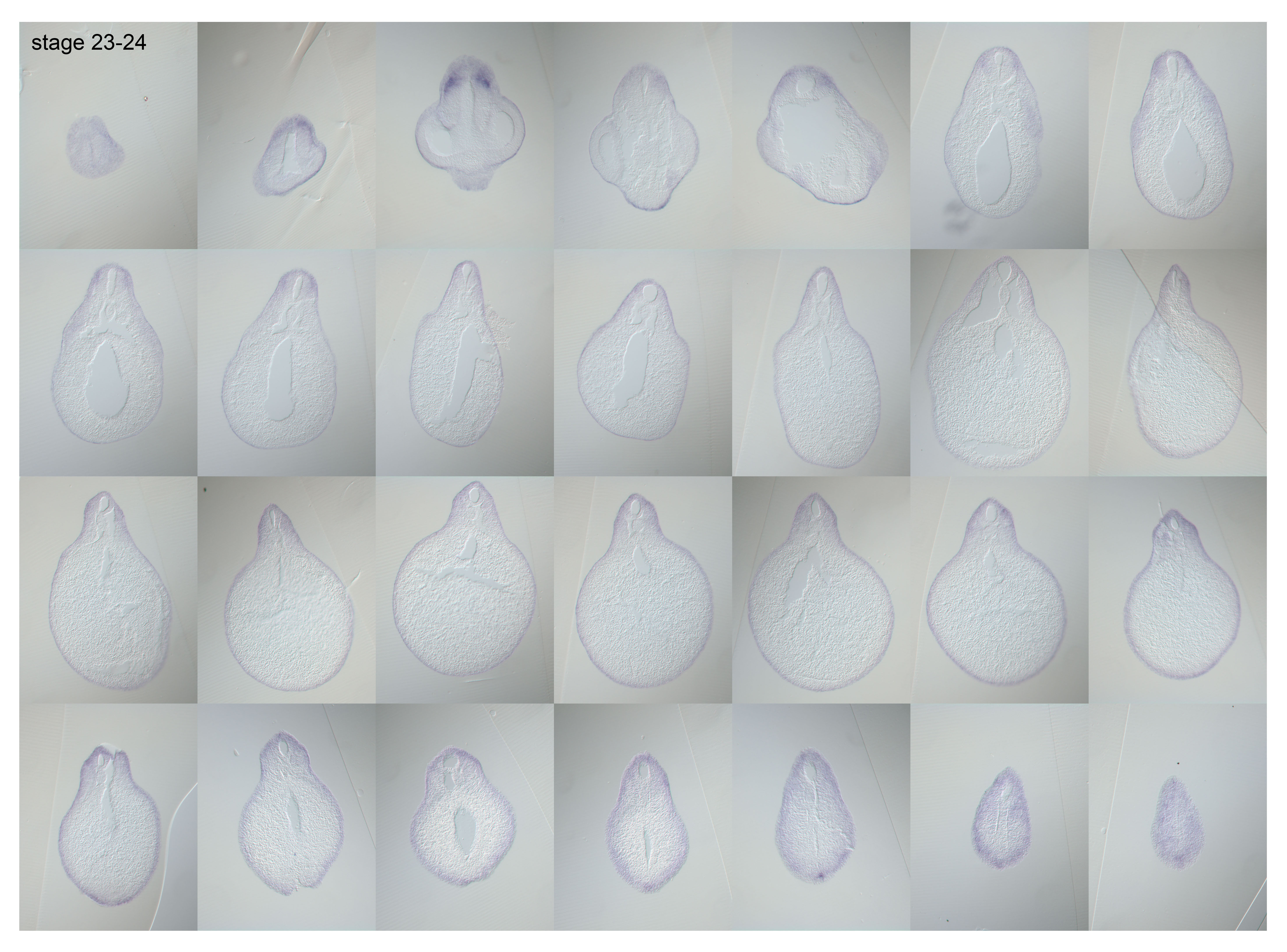

Supplement: S1 File — (Fig A) stage 12.5, (Fig B) stage 14, (Fig C) stage 16, (Fig D) stage 18, (Fig E) stage 19, (Fig F) stage 20, (Fig G) stage 21–22, (Fig H) stage 23–24, (Fig I) stage 24–25, (Fig J) stage 26, (Fig K) stage 28, (Fig L) stage 29–30, (Fig M) stage 31–32, (Fig N) stage 33–34, (Fig O) stage 35–36, (Fig P) stage 38–39, (Fig Q) stage 40. The same embryo has been used to generate all of the images provided at a given stage. (ZIP) [file pone.0193606.s001.zip › S1_File/FigureH-FileS1.jpg]

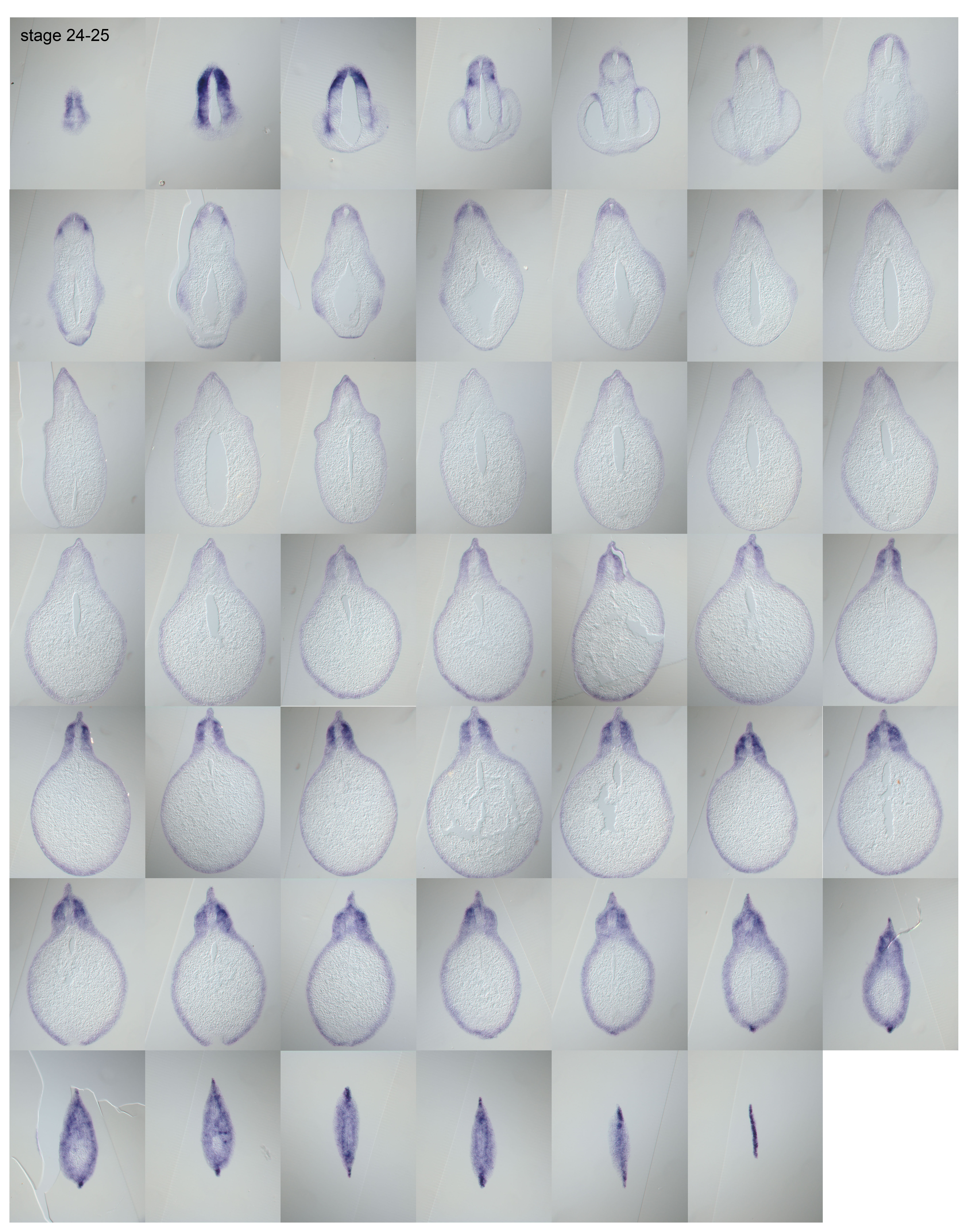

Supplement: S1 File — (Fig A) stage 12.5, (Fig B) stage 14, (Fig C) stage 16, (Fig D) stage 18, (Fig E) stage 19, (Fig F) stage 20, (Fig G) stage 21–22, (Fig H) stage 23–24, (Fig I) stage 24–25, (Fig J) stage 26, (Fig K) stage 28, (Fig L) stage 29–30, (Fig M) stage 31–32, (Fig N) stage 33–34, (Fig O) stage 35–36, (Fig P) stage 38–39, (Fig Q) stage 40. The same embryo has been used to generate all of the images provided at a given stage. (ZIP) [file pone.0193606.s001.zip › S1_File/FigureI-FileS1.jpg]

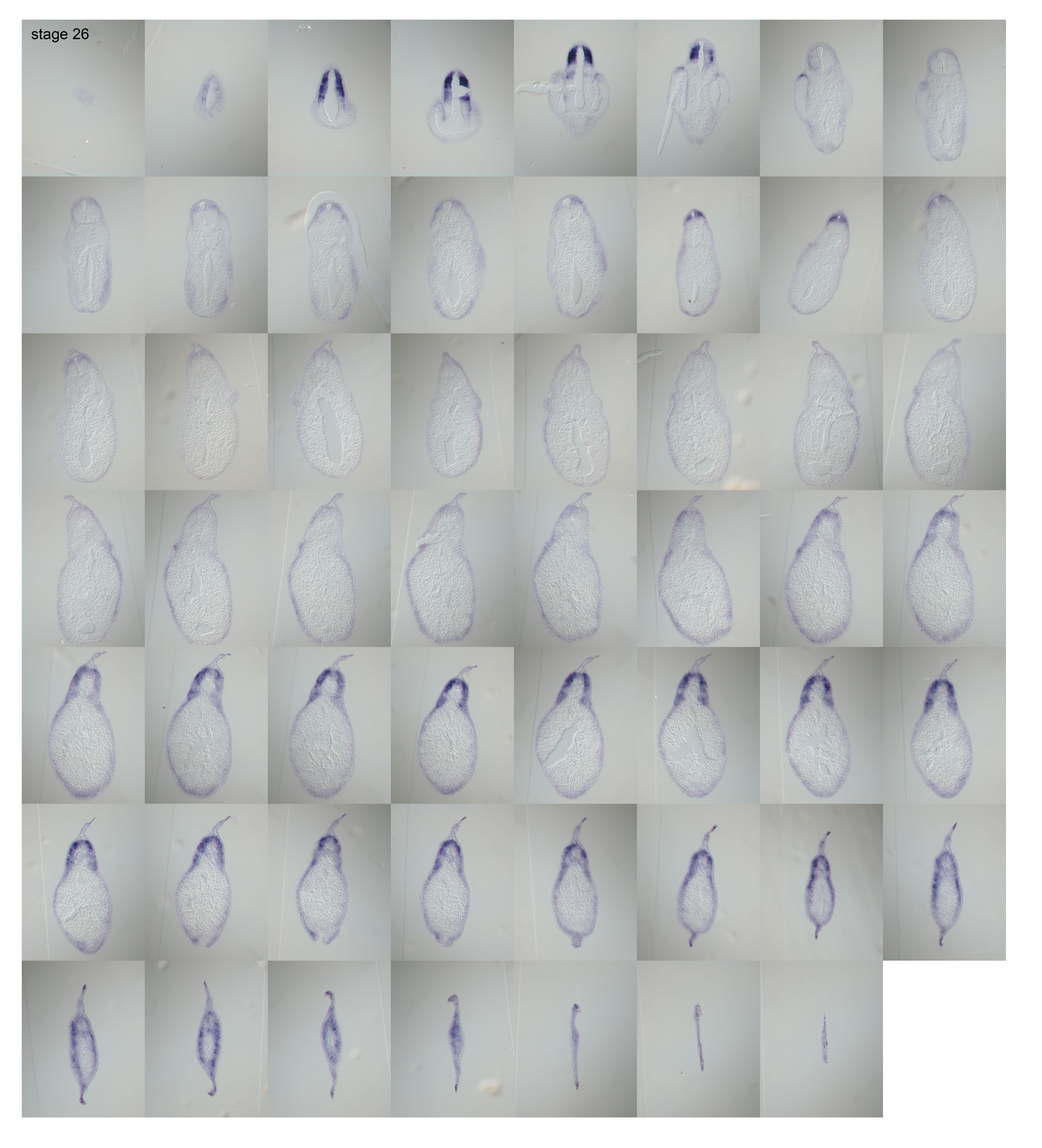

Supplement: S1 File — (Fig A) stage 12.5, (Fig B) stage 14, (Fig C) stage 16, (Fig D) stage 18, (Fig E) stage 19, (Fig F) stage 20, (Fig G) stage 21–22, (Fig H) stage 23–24, (Fig I) stage 24–25, (Fig J) stage 26, (Fig K) stage 28, (Fig L) stage 29–30, (Fig M) stage 31–32, (Fig N) stage 33–34, (Fig O) stage 35–36, (Fig P) stage 38–39, (Fig Q) stage 40. The same embryo has been used to generate all of the images provided at a given stage. (ZIP) [file pone.0193606.s001.zip › S1_File/FigureJ-FileS1.jpg]

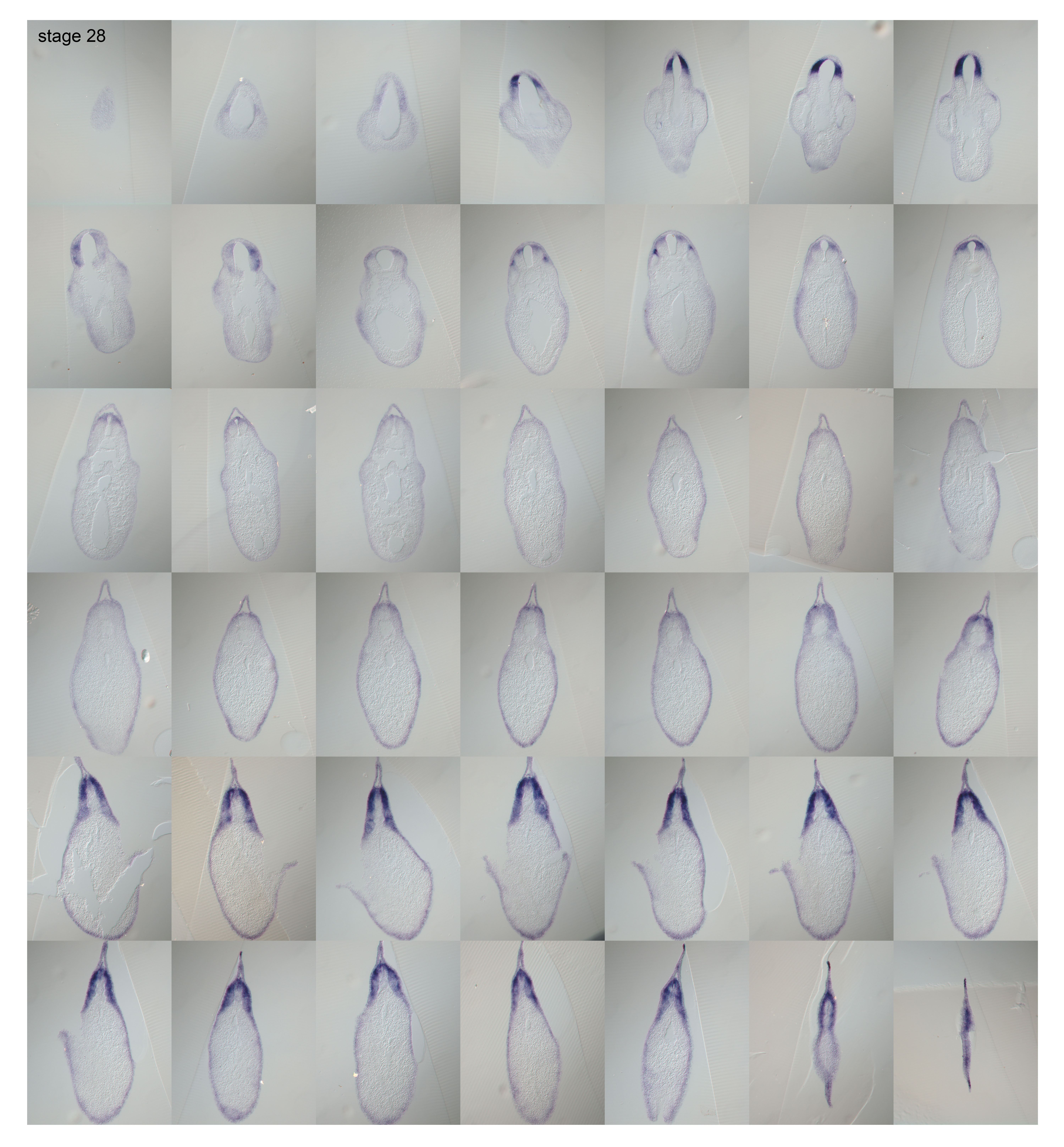

Supplement: S1 File — (Fig A) stage 12.5, (Fig B) stage 14, (Fig C) stage 16, (Fig D) stage 18, (Fig E) stage 19, (Fig F) stage 20, (Fig G) stage 21–22, (Fig H) stage 23–24, (Fig I) stage 24–25, (Fig J) stage 26, (Fig K) stage 28, (Fig L) stage 29–30, (Fig M) stage 31–32, (Fig N) stage 33–34, (Fig O) stage 35–36, (Fig P) stage 38–39, (Fig Q) stage 40. The same embryo has been used to generate all of the images provided at a given stage. (ZIP) [file pone.0193606.s001.zip › S1_File/FigureK-FileS1.jpg]

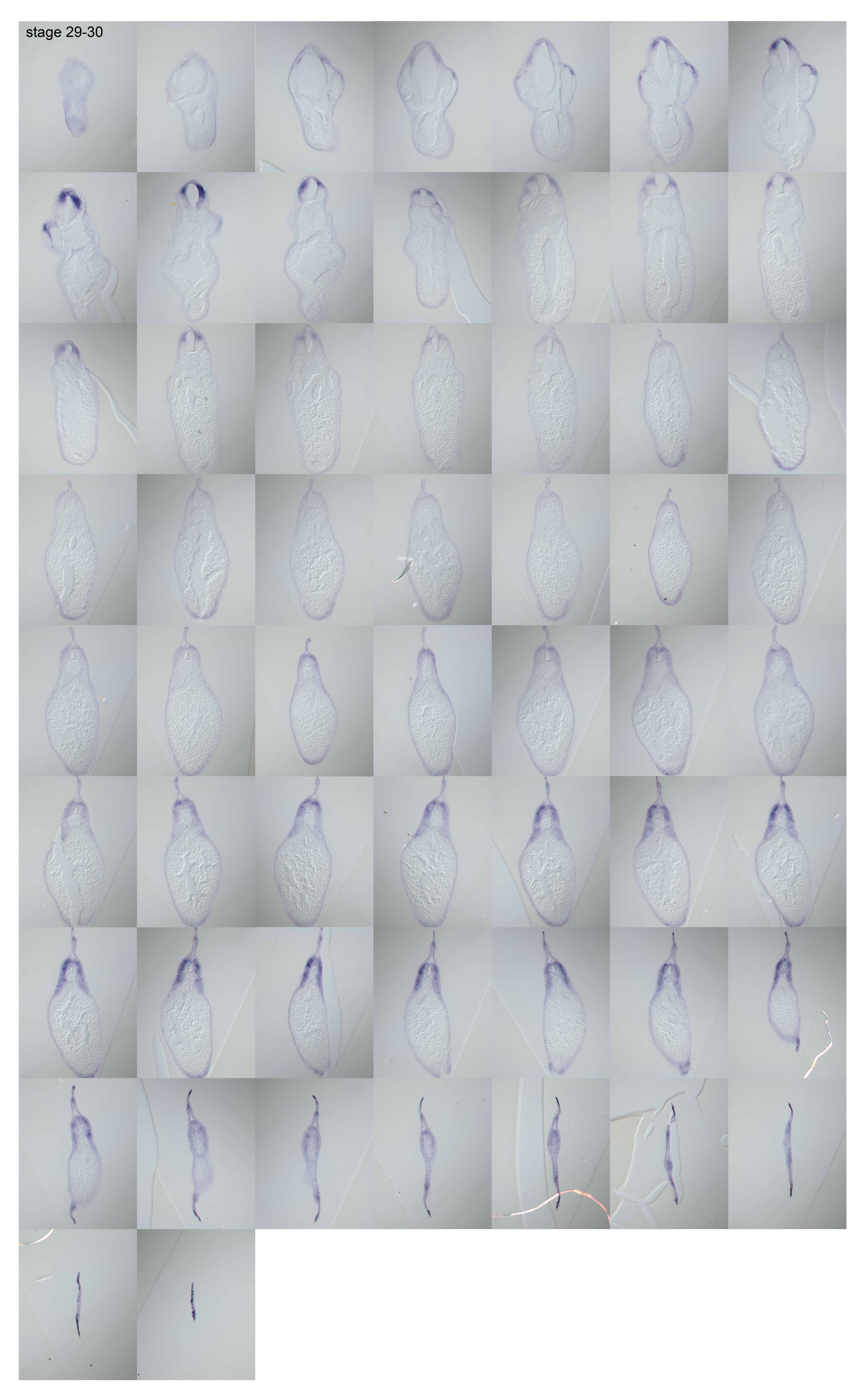

Supplement: S1 File — (Fig A) stage 12.5, (Fig B) stage 14, (Fig C) stage 16, (Fig D) stage 18, (Fig E) stage 19, (Fig F) stage 20, (Fig G) stage 21–22, (Fig H) stage 23–24, (Fig I) stage 24–25, (Fig J) stage 26, (Fig K) stage 28, (Fig L) stage 29–30, (Fig M) stage 31–32, (Fig N) stage 33–34, (Fig O) stage 35–36, (Fig P) stage 38–39, (Fig Q) stage 40. The same embryo has been used to generate all of the images provided at a given stage. (ZIP) [file pone.0193606.s001.zip › S1_File/FigureL-FileS1.jpg]

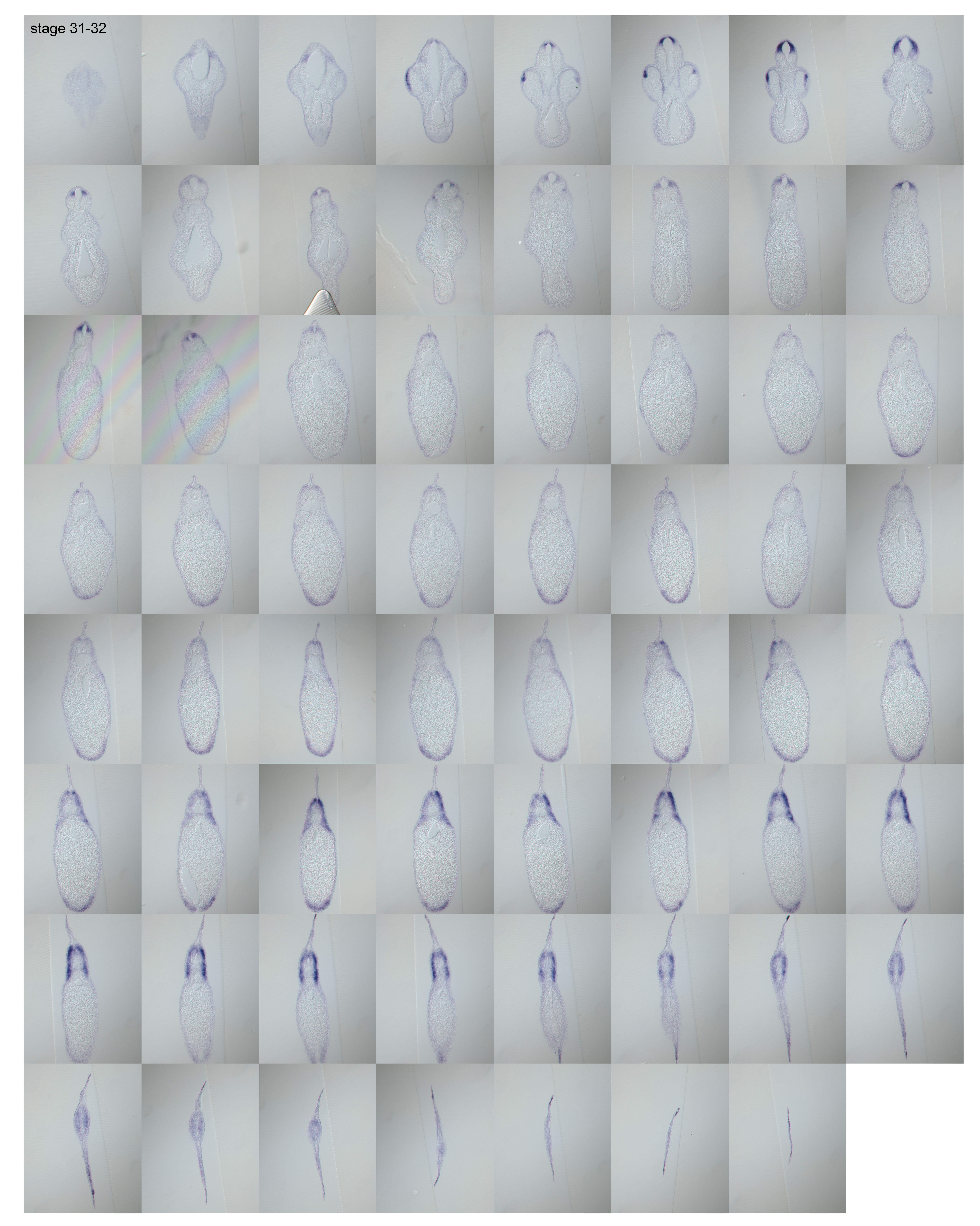

Supplement: S1 File — (Fig A) stage 12.5, (Fig B) stage 14, (Fig C) stage 16, (Fig D) stage 18, (Fig E) stage 19, (Fig F) stage 20, (Fig G) stage 21–22, (Fig H) stage 23–24, (Fig I) stage 24–25, (Fig J) stage 26, (Fig K) stage 28, (Fig L) stage 29–30, (Fig M) stage 31–32, (Fig N) stage 33–34, (Fig O) stage 35–36, (Fig P) stage 38–39, (Fig Q) stage 40. The same embryo has been used to generate all of the images provided at a given stage. (ZIP) [file pone.0193606.s001.zip › S1_File/FigureM-FileS1.jpg]

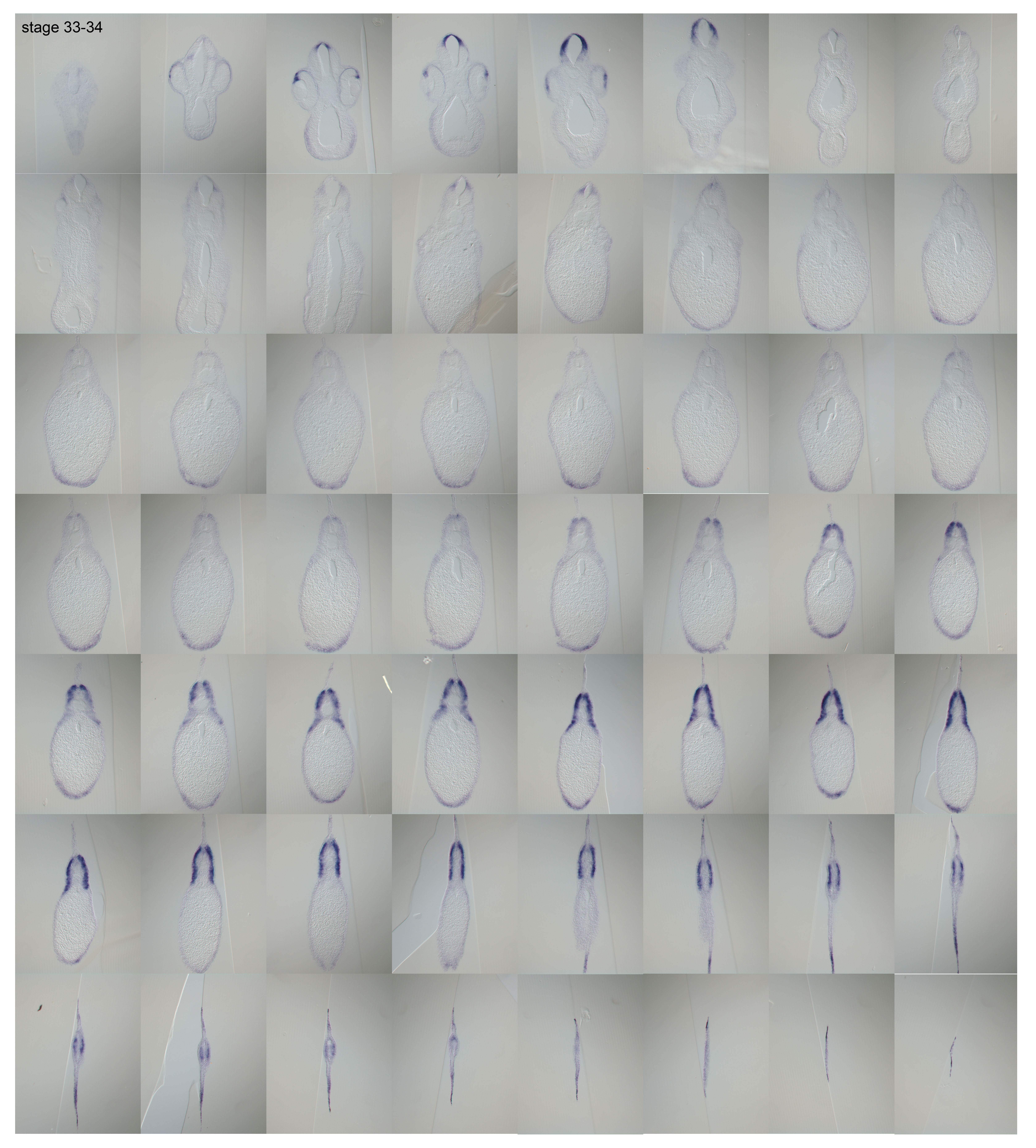

Supplement: S1 File — (Fig A) stage 12.5, (Fig B) stage 14, (Fig C) stage 16, (Fig D) stage 18, (Fig E) stage 19, (Fig F) stage 20, (Fig G) stage 21–22, (Fig H) stage 23–24, (Fig I) stage 24–25, (Fig J) stage 26, (Fig K) stage 28, (Fig L) stage 29–30, (Fig M) stage 31–32, (Fig N) stage 33–34, (Fig O) stage 35–36, (Fig P) stage 38–39, (Fig Q) stage 40. The same embryo has been used to generate all of the images provided at a given stage. (ZIP) [file pone.0193606.s001.zip › S1_File/FigureN-FileS1.jpg]

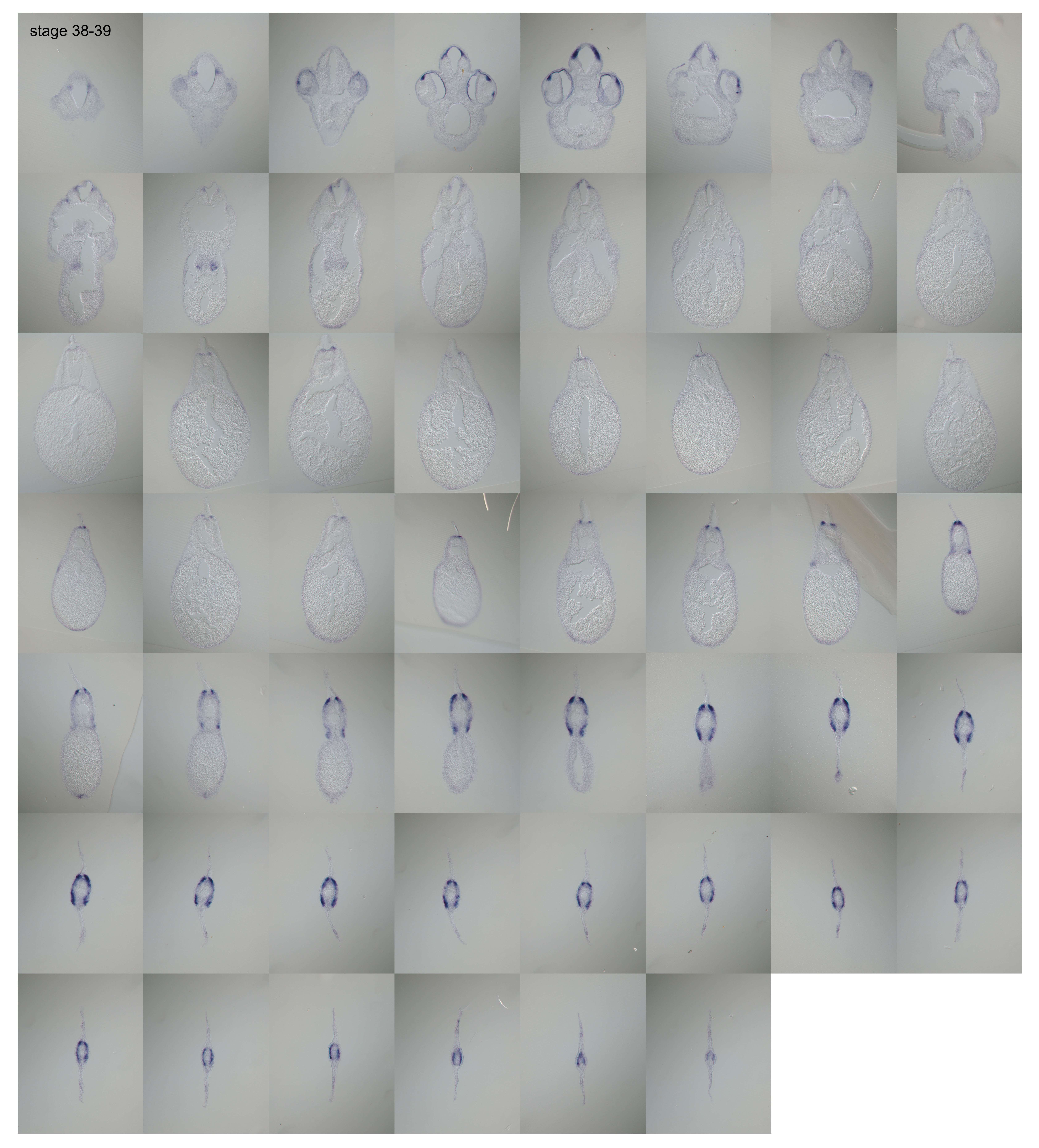

Supplement: S1 File — (Fig A) stage 12.5, (Fig B) stage 14, (Fig C) stage 16, (Fig D) stage 18, (Fig E) stage 19, (Fig F) stage 20, (Fig G) stage 21–22, (Fig H) stage 23–24, (Fig I) stage 24–25, (Fig J) stage 26, (Fig K) stage 28, (Fig L) stage 29–30, (Fig M) stage 31–32, (Fig N) stage 33–34, (Fig O) stage 35–36, (Fig P) stage 38–39, (Fig Q) stage 40. The same embryo has been used to generate all of the images provided at a given stage. (ZIP) [file pone.0193606.s001.zip › S1_File/FigureP-FileS1.jpg]

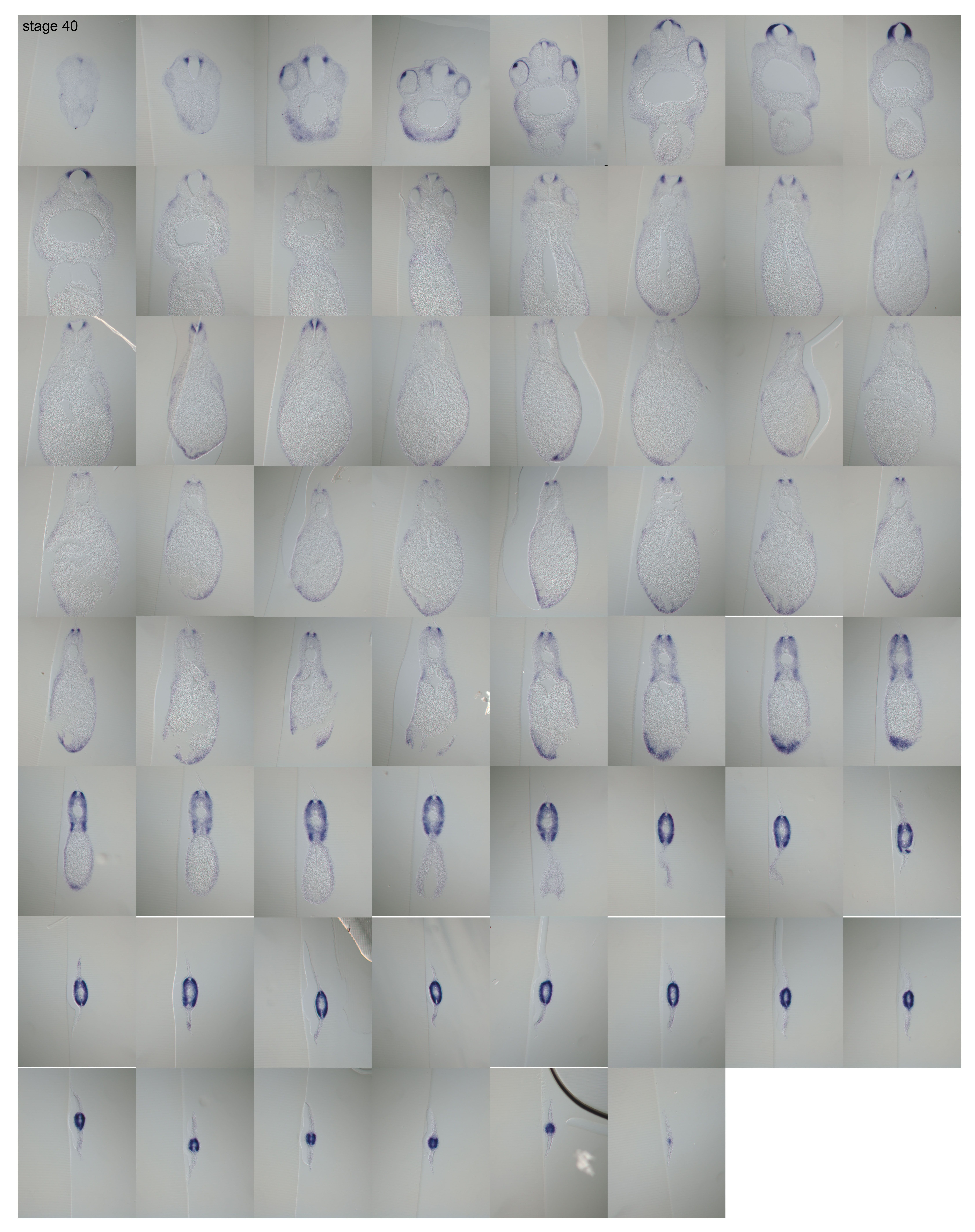

Supplement: S1 File — (Fig A) stage 12.5, (Fig B) stage 14, (Fig C) stage 16, (Fig D) stage 18, (Fig E) stage 19, (Fig F) stage 20, (Fig G) stage 21–22, (Fig H) stage 23–24, (Fig I) stage 24–25, (Fig J) stage 26, (Fig K) stage 28, (Fig L) stage 29–30, (Fig M) stage 31–32, (Fig N) stage 33–34, (Fig O) stage 35–36, (Fig P) stage 38–39, (Fig Q) stage 40. The same embryo has been used to generate all of the images provided at a given stage. (ZIP) [file pone.0193606.s001.zip › S1_File/FigureQ-FileS1.jpg]
